# Non-volatile main memory management methods based on a file system

**DOI:** 10.1186/2193-1801-3-494

**Published:** 2014-09-01

**Authors:** Shuichi Oikawa

**Affiliations:** Faculty of Engineering, Information and Systems, University of Tsukuba, 1-1-1 Tennodai, Tsukuba, Ibaraki, Japan

**Keywords:** Operating systems, Memory management, File systems, Non-volatile (NV) memory

## Abstract

**Abstract:**

There are upcoming non-volatile (NV) memory technologies that provide byte addressability and high performance. PCM, MRAM, and STT-RAM are such examples. Such NV memory can be used as storage because of its data persistency without power supply while it can be used as main memory because of its high performance that matches up with DRAM. There are a number of researches that investigated its uses for main memory and storage. They were, however, conducted independently. This paper presents the methods that enables the integration of the main memory and file system management for NV memory. Such integration makes NV memory simultaneously utilized as both main memory and storage. The presented methods use a file system as their basis for the NV memory management. We implemented the proposed methods in the Linux kernel, and performed the evaluation on the QEMU system emulator. The evaluation results show that 1) the proposed methods can perform comparably to the existing DRAM memory allocator and significantly better than the page swapping, 2) their performance is affected by the internal data structures of a file system, and 3) the data structures appropriate for traditional hard disk drives do not always work effectively for byte addressable NV memory. We also performed the evaluation of the effects caused by the longer access latency of NV memory by cycle-accurate full-system simulation. The results show that the effect on page allocation cost is limited if the increase of latency is moderate.

## 1 Introduction

The upcoming non-volatile (NV) memory technologies, such as phase change memory (PCM), magnetoresistive RAM (MRAM), and spin-transfer torque RAM (STT-RAM), achieve high performance that matches up with dynamic RAM (DRAM) while they provide persistency for data store. They can be used as main memory because of their byte addressability and high performance while they can also be used as secondary storage because of their persistency. Therefore, the integration of their management is possible. Such integration enables the memory allocation for processes and files from the same source, and user application processes can take advantage of its large size by removing the necessity of the page swapping between main memory and storage. Therefore, the integration can improve the system performance. While the advantage brought by the integration were discussed (Bailey et al. 2011; Jung and Cho 2011), there was no research effort to realize it in an actual operating system (OS).

This paper presents the integration methods of the main memory and file system management for NV memory, so that it can be used as both main memory and storage. The presented methods use a file system as their basis for the NV memory management; thus, the internal data management methods of a file system have impacts upon the performance of the integration methods. Therefore, this paper also investigates how these data structures affect the performance.

This paper describes the three methods, direct, indirect, and mmap. The direct method *directly* utilizes the free blocks of a file system by manipulating its management data structures. The indirect method *indirectly* allocates blocks through a file that was created in advance and is dedicated for the use of main memory. The mmap method uses the *mmap* system call for block allocation though memory mapped files. These methods have their own advantages and disadvantages; thus, each method meets different requirements. We implemented these methods in the Linux kernel.

We performed the evaluation of the proposed methods in three phases. Firstly, we performed the preliminary experiment that measures the allocation costs of main memory from a file system. We analyzed the preliminary experiment results and devised the improvements from the analysis. Secondly, we implemented the improvements, and performed their evaluation on a system emulator. The evaluation results of the memory allocation show that 1) the proposed methods can perform comparably to the existing DRAM memory allocator and significantly better than the page swapping, 2) their performance is affected by the internal data structures of a file system, and 3) the data structures appropriate for traditional hard disk drives (HDDs) do not always work effectively for byte addressable NV memory. In the first and second phases, we disregarded the performance difference between NV memory and DRAM because our focus was the integration of the main memory and file system management. Finally, we performed the evaluation of the effects caused by the longer access latency of NV memory by cycle-accurate full-system simulation. The results show that the effect on page allocation cost is limited if the increase of latency is moderate. To the best of our knowledge, we are among the first to design and implement the integration methods of the main memory and file system management for NV memory and also to perform their evaluation. Note that wear leveling is outside the scope of this paper while it can be performed at the software or hardware level; thus, the proposed methods do not consider it.

While this paper is an extension to a previous paper published at SEUS 2013 (Oikawa 2013), it provides the detailed description of its background work along with two new evaluation results, one evaluates the effects on application performance and the other evaluates the effects caused by the access latency of NV memory.

The rest of this paper is organized as follows. Section 2 describes the background of the work. Section 3 proposes the integration methods, and presents their design and implementation. Section 4 shows the preliminary experiment results, and describes their analysis. Section 5 describes the improvements, and shows their experiment results. Section 6 evaluates the effects on application performance. Section 7 evaluates the effects caused by the access latency of NV memory. Section 8 describes the related work. Finally, Section 9 concludes this paper.

## 2 Background

This section describes the new NV memory technologies and the Linux file system infrastructure as the background of the work.

### 2.1 NV Memory

Upcoming NV memory devices enable high performance along with persistent data store with no power supply. PCM, MRAM, and STT-RAM are such examples. The most major memory devices are currently DRAM and flash memory, which use electrical charge to memorize binary information. Since the certain capacity for electrical charge needs to be maintained for memorization, there are the limitations of scaling DRAM and flash memory. DRAM also has the other power consumption problems, such as leakage and refresh dynamic power. Therefore, NV memory is considered to be a candidate to replace or to be used along with DRAM and flash memory.

The new NV memory devices use resistance values instead of electrical charge; thus, they can maintain data without power supply and provide persistency. PCM technology utilizes a chalcogenide material. Since it takes the two states, amorphous and crystalline, and they take different resistance values, it can be used to memorize binary information. Changing a value means the transformation of the material state; thus, the transformation takes the time to do so, and also it shortens its lifetime. MRAM technology utilizes a magnetic tunneling junction (MTJ). Two ferromagnetic layers and one tunnel barrier layer compose an MTJ. One of the two ferromagnetic layers takes different magnetic directions, and different directions makes different resistance values; thus, different magnetic directions can be used to memorize binary information. STT-RAM is a newer version of MRAM, and performs comparably with DRAM. Such high performance makes it possible to use it in the same manner as DRAM (Park 2012). While MRAM and STT-RAM are more suitable for main memory than PCM because of their higher performance and endurance, they are less dense; thus, PCM is more suitable for the uses that require large capacity.

When these NV memory technologies emerged, the active investigations of their use for either main memory or secondary storage were performed. PCM was first considered to be a candidate to replace DRAM since its development was more advanced than MRAM and STT-RAM. When PCM emerged to be practical as a memory device, active researches were conducted to utilize it as main memory by mitigating its limitations (Lee et al. 2009; Qureshi et al. 2009, 2010; Zhou et al. 2009). These researches investigated the aspect of computer architecture, and there is no or only a minor role of the operating systems (OSes) (Mogul et al. 2009; Zhang and Li 2009). Independently from these computer architecture researches, there were some researches that investigated the aspect of OSes. The investigations on the construction of file systems on NV memory were conducted in order to take advantage of its byte addressability and larger capacity (Condit et al. 2009; Wu and Reddy 2011).

### 2.2 Linux file system infrastructure

**Figure 1:**
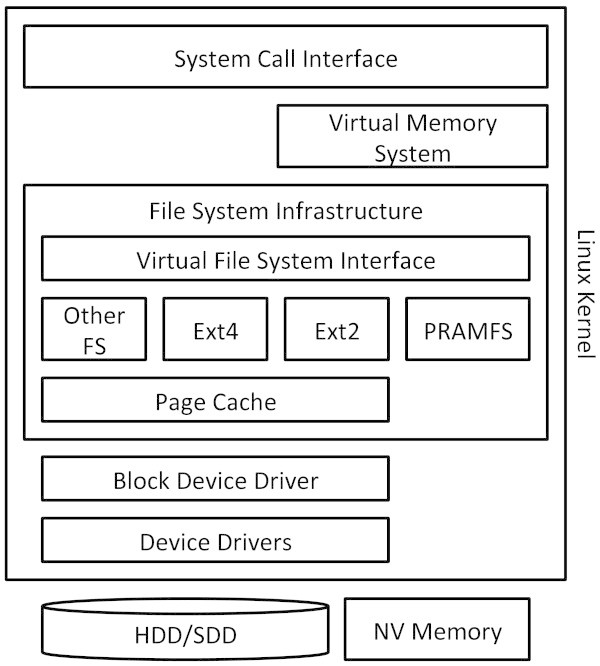
**Overview of the Linux file system infrastructure.**

The file system infrastructure of the Linux kernel is designed to accommodate various types of file systems that interact with different kinds of storage devices. Figure 1 depicts the overview of the Linux file system infrastructure.

User processes interact with a file system through the system call interface. There are basically two kinds of the system call interface to deal with a file. One directly interacts with a file, and the other one goes through the virtual memory system. The former includes the traditional read and write system calls, and the latter includes the mmap system call. There can be multiple file systems inside the single Linux kernel. They export the common interface, and are managed under the virtual file system (VFS) interface. Therefore, the system call interface and the virtual memory system do not need to take account of the differences of the internal implementation of file systems. The page cache mechanism is employed to interact with block devices. Since data on block devices needs to be transferred to memory for processors to access it, the mechanism allocates physical memory pages to cache such data. The mechanism manages them literally at the page size, so that they can also be utilized directly by the virtual memory system. The block device driver is the common layer to interact with individual device drivers for storage devices, and individual device drivers deal with storage devices for their types.

There are memory-based file systems, such as PRAMFS (Protected and Persistent RAM Filesystem 2012) in the figure. Such a file system does not require the page cache mechanism and device drivers. Because processors can directly interact with memory used as its storage, its code simply uses memory access instructions to read and write data on it. It is also possible for a device driver to interact with memory used as its storage. A ramdisk driver is such an example. When a ramdisk driver is used, traditional, block-based file systems can use memory as their storage. In this case, however, there is access overhead incurred by the page cache mechanism and the block device driver.

A typical scenario to read data from a file system is the following. When a user process issues the read system call to access data, the corresponding function in the system call interface is invoked. It calls the virtual file system interface, and then calls the file system that corresponds to the file that is being read. If the file system is a block-based file system, it accesses its management data structure to find out the block number where the data resides. If the management data structure is not in the page cache, it needs to be read from storage through the device drivers. Once the file system finds the block number, the data on it is transferred to the page cache through the device drivers. The data in the page cache is finally copied to the buffer of a user process. If the file system that is being accessed is a memory-based file system, both of the management data structure and the designated data can be directly accessed without the interventions of the page cache and device drivers. Therefore, it is far much simpler for a memory-based file system to access data on it.

The integration of the virtual memory system and file systems are described later in Section 3.2.

## 3 Design and implementation

This section describes the design and implementation of the integrated main memory and file system management for NV memory. The implementation is described for the Linux kernel.

### 3.1 Target system structure

**Figure 2:**
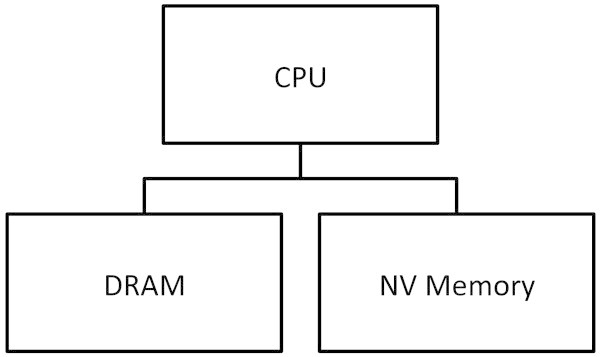
**Target system memory structure.**

**Figure 3:**
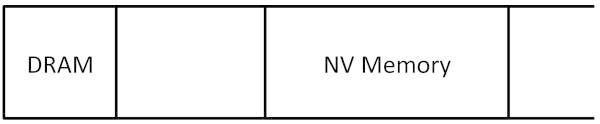
**Physical address space of the target system.**

Computer systems that equip with NV main memory are not yet available publicly as commercial products. Since there is no typical architecture that we can model, we design an architecture that are realistic and practical as a target system. In this paper, 1) we employ NV memory and DRAM to form main memory of the target system, and 2) we place both of them in the same physical address space. CPUs access DRAM and NV memory though their memory bus(ses) with appropriate physical addresses; thus, the same memory access instructions are used to access them. Figures 2 and 3 depict the memory architecture and physical address map of the target system.

Our goal is to establish the integrated management of main memory and storage. Since NV memory is used as main memory and storage, it takes the both roles. The integration methods proposed in this paper utilize a file system, which is created on NV memory, as a basic management mechanism. A file system manages data blocks that store directories and files, and is designed to maintain the data across the system shut down and start up. The integration methods have the free blocks of the file system temporarily used for main memory. Because the kernel manages NV memory, direct access to it from user applications is prohibited unless its certain pages are mapped to their virtual address spaces for the use of main memory. Therefore, the internal state of the file system is protected from user applications.

We consider that the architecture described above is realistic and practical as a target system. Because we primarily target mobile devices, such as note PCs, tablets, and smart phones, to apply the proposed methods, NV memory can meet the requirements of storage capacity and also take more advantage of its power efficiency. While NV memory can constitute the whole main memory of a system, DRAM is also useful to hold data regions, which are known to be volatile and overwritten soon. Examples of such regions include stacks and buffers used for data transfer. Therefore, it should be reasonable to consider that a target system’s memory consists of both DRAM and NV memory.

We employ the QEMU system emulator and the MARSSx86 cycle-accurate simulator (Patel et al. 2011) to construct the target system described above and to execute Linux on it. The more details of the emulation and simulation environments are described in Section 4.1 and 7.1, respectively.

### 3.2 Virtual memory system

**Figure 4:**
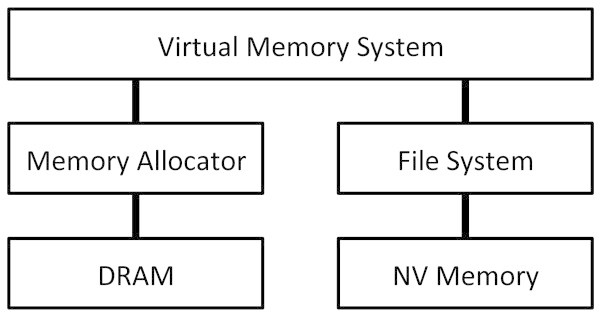
**Virtual memory system architecture and its relationships with the memory allocator and a file system.**

This section describes the virtual memory system architecture of the Linux kernel. The virtual memory system is constructed on the memory allocator, which manages DRAM, and file systems, which manage storage. They work together to make the virtual memory system efficient and flexible. Figure 4 depicts the virtual memory system architecture. Since storage is NV memory for our target system, a file system manages NV memory. When physical memory pages need to be allocated from DRAM, the virtual memory system consults the memory allocator. After the allocation is successful, it inserts the allocated pages in a page table to map them in a virtual address space. The allocated pages finally become accessible.

XIP^a^-enabled file systems, such as Ext2 and PRAMFS (Protected and Persistent RAM Filesystem 2012), make it possible to directly map their files in user process address spaces. In this case, allocated file system blocks are used as physical memory pages, and they are directly mapped into a virtual memory address space. Since direct access to the physical memory pages of files is possible, no copying of pages between DRAM and NV memory is necessary. Ext2 and PRAMFS are the only XIP-enabled file systems that support both read and write. We chose PRAMFS as our target file system to implement the integrated memory management methods because it was literally designed for the use on NV memory and does not require the block device driver.

### 3.3 Integrating main memory and file system management

This section describes the integrated memory management methods, by which NV memory can be used for the memory allocation for both processes and files. Since NV memory is managed by a file system, its physical memory pages that will be used as main memory also need to be allocated from a file system. Such pages are, however, usually allocated from the memory allocator; thus, there needs to be a path for the memory allocator to allocate pages from a file system. Therefore, in Figure 4, the memory allocator and a file system need to be connected to create a link between them. Such a link was originally not connected. The memory allocator could only allocate the pages of DRAM without connecting the missing link, but it worked just fine because DRAM was only memory that could be used as main memory. When NV memory can be used as main memory and is under the management of a file system, connecting this missing link is necessary for the memory allocator to allocate the pages of NV memory and to use them as main memory.

In the Linux kernel, __alloc_pages_nodemask() is the function that the memory allocator calls to allocate pages, and free_hot_cold_page() is called to free allocated pages. The existing implementation only deals with DRAM; thus, they were modified to call the page allocation and freeing functions of a file system. The three methods described in the next section implement these functions for the page allocation and freeing. After the modification, when the kernel tries to allocate pages, __alloc_pages_nodemask() first consults a file system by calling the allocation function provided by the one selected among the proposed methods. It searches available free pages of a file system and returns them for the use of main memory. The returned pages were removed from the pool of free pages in order to avoid them to be used in a file system. When pages allocated from NV memory are being freed, free_hot_cold_page() return them to a file system by calling the freeing function of the proposed method. It returns them to the pool of free pages in order to make them available for future use.

There are a few implementation issues that should be considered. First, the granularity of memory pages allocated by __alloc_pages_nodemask() needs to be the same as the native page size of the processor architecture. If the block size of a file system is different from the native page size, it is necessary to find consecutive free blocks, of which size and alignment match the native page size. Therefore, the native page size of the processor architecture needs to be used as the block size of the file system for efficiency and simplicity.

Second, __alloc_pages_nodemask() takes gfp_mask as one of its arguments, and gfp_mask specifies the attributes that allocated pages need to satisfy. If GFP_KERNEL is specified, the allocated pages can be used within the kernel. Although there is no problem with using the pages, which were allocated from a file system, within the kernel, some of these pages can be still in use when a system halts because such an allocation policy does not cause any problems when only DRAM, which is volatile, is used as main memory. Depending upon the method used for the page allocation from a file system, such pages that are not freed can make a file system inconsistent. If GFP_HIGHUSER_MOVABLE is specified, the allocated pages are used only for user processes. In this case, a file system will not be left inconsistent since all processes are killed and all of the pages allocated for them are returned when a system halts. Therefore, the usage of the pages from a file system needs to be adjusted depending upon the used page allocation method.

The third issue is the identification of the pages allocated from a file system. The pages allocated from DRAM need to be returned to the DRAM memory allocator, and those from a file system need to be returned to it. When free_hot_cold_page() is called to free pages, however, there is no distinction between those from DRAM and a file system. free_hot_cold_page() has to identity where they are allocated from; thus, there must be a means to realize it. Therefore, a new attribute NVmemory is added to identify the pages allocated from a file system. When the pages with NVmemory attribute are freed, they are returned to a file system.

### 3.4 Proposed methods for integration

This section describes the three methods, direct, indirect and mmap, for the integrated memory management. These methods connect the missing link between the memory allocator and a file system since they use a file system as their basis for the NV memory management. The direct method directly utilizes the free blocks of a file system by manipulating its management data. The indirect method indirectly allocates blocks through a file that was created in advance and is dedicated for the use of main memory. The mmap method uses a memory mapped file for the block allocation of a specific memory region. These methods have their own advantages and disadvantages; thus, each method meets different requirements. The details of these methods are described below.

#### 3.4.1 Direct method

The direct method takes free blocks that are managed by a file system for files, and allocate them for the use of main memory. This method consults the management data structures of a file system in order to find and remove free blocks; thus, this method requires the *direct* manipulation of the internal data structures of a file system. The manipulation requires the implementation of additional code.

The advantage of the direct method is the tight integration of main memory and file system management. Any of the free blocks of a file system can be used for both main memory and files. The use of the free blocks is not determined and they are remained free until their allocation; thus, this method does not waste the free blocks. The disadvantages are the dependency on the implementation of a file system and the crash recovery. The dependency issue involves two aspects, the effect of the internal data structures to the performance and the implementation cost of the additional code. The crash recovery is required because the allocated blocks for the use of main memory do not belong to any file and there can be no reference to them from anywhere; thus, these blocks cause the inconsistency of a file system when a system abnormally crashes and they are not correctly freed. By maintaining the information of the pages allocated for the use of main memory, these pages can be reclaimed correctly on the next start up. It is our future work, though.

#### 3.4.2 Indirect method

The indirect method utilizes the blocks of a file that was created in advance and is dedicated for the use of main memory. This method does not need to consult the management data structures of a file system, but *indirectly* uses the blocks that were allocated for a file. When a system boots up, the content of the file is initialized by creating a linked list of its blocks; thus, all of the blocks of the file need to be allocated when created. The block allocation for main memory is done by simply taking blocks out of the linked list. When they are freed, they are added to the linked list.

The advantages of the indirect method are contrary to the direct method. The indirect method does not depend on the implementation of a file system. The provision of the XIP feature of a file system requires the implementation of get_xip_mem() interface. This interface converts a file offset to its virtual address in the kernel and its page frame number. By using this interface, it is possible to access all of the blocks of a file used for main memory, and its initialization can be done independently from the internal implementation of a file system. The linked list of the free blocks also makes the allocation cost independent from the internal implementation. Moreover, since the blocks used for main memory are allocated for a file, they do not cause the inconsistency of a file system when a system crashes. The disadvantage is that the blocks of a file used for main memory are preallocated; thus, their use is fixed for main memory. It is desirable to adjust the size of the preallocated file. While it is easy to extend the file size as more blocks need to be allocated for main memory, more work is necessary to shrink it.

#### 3.4.3 Mmap method

The mmap method uses the mmap system call to create the mapping of a file in the virtual address space of a user process. While this method is not new in terms of the mechanism, it is provided as an alternative method to access files. This paper proposes the mmap method as a mechanism to allocate blocks from a file system for the use of main memory. By employing XIP-enabled file systems, the allocated blocks can be directly mapped in the virtual address space of a process. The allocation is done by mapping a file of a necessary size in the virtual address space of a process. The actual allocation of blocks for the file is done by writing data in the mapped region. If there is no block allocated at the address where data is written, a new block is allocated. Before mapping a newly created file, its size must be set by using the truncate or ftruncate system call. Otherwise, accessing the mapped region causes an error.

The advantages of the mmap method are no necessity of modification to the kernel and its concrete integration of a single memory region and a single file. Linux and also other modern operating systems implement the mmap system call natively; thus, the mmap method does not require any modifications to the kernel, nor the file system driver. Date written to memory is directly reflected in the mapped file, and no synchronization is needed to make memory and the mapped files coherent; thus, a user process can always persistently store data in the mapped file. This means that the mmap method can be a basis to facilitate the access to NV memory and potentially persistent storage by providing a simple library. The mmap method also provides the advantages of the indirect method, no dependency on the implementation of a file system and the consistent file system state when crashed. The disadvantages are its page allocation performance and less transparent usage. As shown later in Section 4 and 5, the allocation cost of the mmap method is worse than the direct and indirect methods. Even after the improvements described in Section 5, the allocation cost of the mmap method is more than two times as much as those of the direct and indirect methods. The allocation cost has, however, a limited impact on the overall performance of a system when the mmap method is desired. If a process needs to store data persistently in a mapped file, it is very likely that the file is kept mapped during its execution; thus, the allocation of a certain block happens only once. The mmap method is less transparent to applications because it requires them to specifically use the mmap system call or the malloc library function needs to be modified to use it.

### 3.5 Comparison of proposed methods

This section compares the proposed methods for the integrated memory management. Table 1 summarizes their advantages and disadvantages. It is apparent that the advantage and disadvantages of the direct method are contrary to the indirect method. The only essential disadvantage of the direct method is, however, its high implementation cost. The performance and inconsistency issues can disappear if a file system that are optimized for the integrated memory management is used. Therefore, the choice between the direct and indirect method can be made based on the trade-off between the utilization of free space of a file system and implementation cost, of which importance can be further affected by the capacity of NV memory storage. If the capacity of NV memory storage is limited, the maximum and flexible utilization of free space of a file system is more important than the implementation cost; thus, the direct method is better for this case. In contrast, if the capacity of NV memory storage is large and there is plenty of free space, it is possible to opt for the indirect method because the maximum utilization of free space is not necessary.

**Table 1:** **Comparison of the proposed methods for the integrated memory management**

Methods	Advantages	Disadvantages
Direct	Maximum and flexible utilization of free space of a file system.	1) High implementation cost (implementation is necessary for each file system). 2) File system dependent performance. 3) Possible inconsistency of a file system when crashed.
Indirect	1) Low implementation cost (implementation is independent from file systems). 2) File system independent performance. 3) Consistency of a file system when crashed.	Inflexible allocation of file system spaces used for main memory.
mmap	1) Flexible utilization of free space of a file system. 2) Low implementation cost (no modification is necessary for the kernel). 3) Consistency of a file system when crashed.	1) File system dependent performance. 2) Less transparent usage for memory allocation.

The mmap method has the characteristic that is a mixture of the direct and indirect methods. Even if a file system is optimized for the integrated memory management, it is difficult for the mmap method to match the direct and indirect methods for memory allocation performance because a page fault is an only means for page allocation. Furthermore, the mmap method is not transparent to applications because they need to be modified to directly use it. This issue can be resolved by employing a modified malloc library function as described in Section 6.

## 4 Preliminary evaluation and analysis

This section first describes the evaluation method, and then shows the preliminary experiment results that measure the allocation costs of main memory from a file system.

### 4.1 Evaluation method

We developed the target system architecture described in Section 3.1 based on the QEMU system emulator. The QEMU version 1.0.1 was used for the measurements of the evaluation, and QEMU emulates the x86_64 instruction set architecture. We modified QEMU to incorporate the emulation of NV memory that persistently maintains its contents across the shut down and start up of the emulator. The contents of emulated NV memory are stored in a file to maintain its persistency. QEMU maps the file into its emulated physical address space. When QEMU emulates NV memory, the following option is specified:


QEMU invoked with the above option maps the file emulating NV memory (nvm.img) from 0x100000000 of the physical address. The size of NV memory is the same as the file emulating NV memory. The file size used for the evaluation is 4 GB. The experiments described in this section were performed with 128 MB DRAM along with the NV memory; thus, the -m 128 option is also specified. QEMU passes the DRAM size to BIOS, and the Linux kernel recognizes it. The information of NV memory is, however, not passed in order to have the Linux kernel manage it separately from DRAM. The modified QEMU executes the Linux kernel that includes the modifications of the proposed methods. The version of the Linux kernel modified and used for the experiments is 3.4.

We use the number of instructions executed as the measure of execution costs instead of execution times since times counted by interval timer interrupts from a clock device are not reliable on system emulators. When QEMU is invoked with the -icount 0 option, it enables the time stamp counter (TSC) register to count the number of instructions executed. The RDTSC instruction is used to read the TSC value.

The following evaluation results presented in this section do not take into account the difference of access latencies between DRAM and NV memory, and they are treated the same.

### 4.2 Page allocation costs

This section shows the preliminary measurement results of the allocation costs of main memory pages from a file system and compares them with those that use the memory allocator and the page swapping mechanism. Integrating the main memory and file system management on NV memory brings a large amount of physical memory to user processes; thus, such integration should be able to remove the necessity of the paging swapping and to achieve the performance improvement to allocate a large amount of memory. In order to verify the effectiveness of the integration, we executed a program that allocates a memory region by using malloc() and performs writes to the beginning of the page boundaries for the actual allocation of physical memory pages. These writes are necessary for the actual allocation of pages. For the mmap method, the mmap system call is used to allocate a memory region, instead.

**Figure 5:**
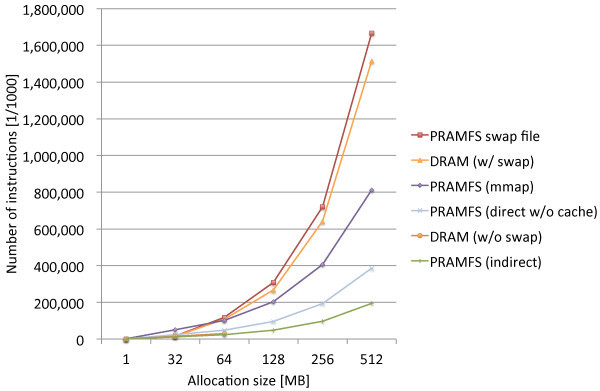
**Preliminary comparison of page allocation costs.**

The measurements were performed for several cases including the three proposed methods. Figure 5 shows the results. In the figure, DRAM (w/o swap) and DRAM (w/ swap) are the cases that only DRAM is used as main memory without and with a swap device, respectively. For DRAM (w/ swap), a swap device is a ramdisk created on NV memory. PRAMFS (direct w/o cache), PRAMFS (indirect), and PRAMFS (mmap) are the cases that PRAMFS is constructed on NV memory and the direct, indirect, and mmap methods are used for main memory allocation from the file system, respectively. PRAMFS swap file is the case that only DRAM is used as main memory, and a swap file created on PRAMFS is used as a swap device. The all blocks of the swap file is allocated in advance.

Firstly, the measurement results apparently show the efficiency of the memory allocation from NV memory and the inefficiency of the memory allocation that involves page swapping. DRAM (w/ swap) and PRAMFS swap file are approximately 7.8 and 8.1 times as much as the indirect method, respectively. Although it is slightly faster to use a ramdisk than PRAMFS, paging swapping adds a significant amount of overheads for memory allocation. Therefore, integrating the main memory and file system management can improve their performance when they require a large amount of physical memory.

Among the three proposed methods, the indirect method performs the best. The direct method comes next to the indirect method with 98% overhead for the allocation of 512 MB of memory. The cost of the mmap method is approximately 4.2 times as much as that of the indirect method. The indirect method is different from the direct and mmap methods because it creates the linked list of the free blocks, which makes the allocation cost independent from the internal implementation of the file system. Both the direct and mmap methods depend on the internal implementation for block allocation; thus, their allocation costs also depend on it. Therefore, there must be the interrelationship of the the block allocation costs between the file system’s native operations and the proposed methods except for the indirect method.

### 4.3 Comparison of file system performance

This section investigates the interrelationship of the the block allocation costs between the file system’s native operations and the proposed methods. Blocks on a file system are allocated by creating a file and writing data in it; thus, we executed a program that performs these operations. We executed the program also on Ext2 constructed on NV memory for comparison. Since Ext2 cannot directly access NV memory, a block device driver of NV memory was implemented and is used as its base device. The device driver implements the necessary function to enable the XIP feature of Ext2.

**Figure 6:**
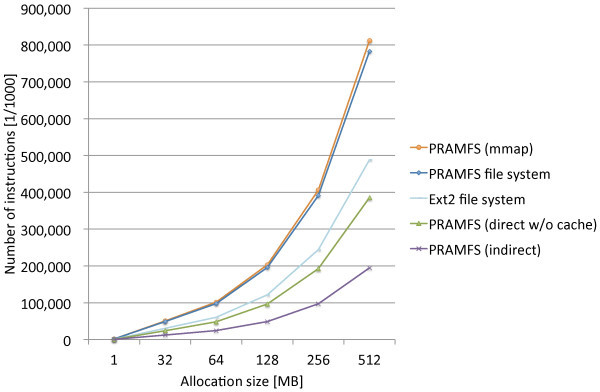
**Comparison of page allocation costs of file systems.**

Figure 6 shows the measurement results along with those of the proposed methods. PRAMFS file system and Ext2 file system are the cases that the program creates a file and writes a byte at the beginning of each page boundary to allocate the specified amount of blocks. These are basically the same operations performed to measure the page allocation costs of the proposed methods except that they use different system calls.

The measurement results show the direct interrelationship between the mmap method and writing to a file since their allocation costs are very close. They basically use the same set of the internal functions that PRAMFS implements for the block allocation of a file. The results also show the deficiencies of PRAMFS in terms of allocating blocks since the result of Ext2 is much better.

Both PRAMFS and Ext2 employ the bitmap data structure to distinguish allocated and free blocks. The bitmap structure can be a good solution to manage free blocks for HDDs because it makes it easy to find a free block that is close to another one and helps to make the allocated blocks of a file as contiguous as possible. While contiguous block allocation is important for rotating HDDs to efficiently access files, it adds too much cost for NV memory because bitwise operations to find a clear bit in the bitmap structure are significantly time consuming. Therefore, the free block management of a file system on NV memory requires an alternative mechanism.

One of the reasons why Ext2 performs better for block allocation than PRAMFS can be because of its block preallocation feature. When a new block is allocated, Ext2 internally preallocates several blocks adjacent to the block just allocated. Such preallocation helps to make the blocks of a file more contiguous and also to make the subsequent block allocation much more efficient; thus, Ext2 can perform better for block allocation than PRAMFS.

These analysis results give us hints for the improvements that can be made to PRAMFS.

## 5 Improving a file system for non-volatile main memory

This section describes the two improvements of a file system devised from the analysis results described in the previous section. The improvements are applied to PRAMFS, and their evaluation results are shown below.

### 5.1 Block preallocation cache

The first improvement is the block preallocation cache, which is hinted by the preallocation feature of Ext2. Ext2 preallocates blocks to reduce the overhead to search a clear bit in the free block bitmap that manages free blocks. It uses a preallocated block for the subsequent block allocation, and avoids searching a free block for each block request. If bitwise operations can be totally avoided, the overhead can be further reduced.

The block preallocation cache is a linked list of the free blocks preallocated from a file system. In response to a block allocation request, a block is taken out from the linked list instead of searching a clear bit in the free block bitmap. When the list is empty, 64 blocks are allocated together from a file system and added to the list. Searching 64 free blocks in the bitmap can avoid bitwise operations. Since our target CPU is x86_64, its word size is 64-bit. By considering the bitmap as an array of 64-bit elements, searching 64 free blocks is simply searching an array element of which content is 0. When a block is freed, it is returned to the block preallocation cache. If there is an enough number of blocks in the cache, the freed block is returned to a file system.

**Figure 7:**
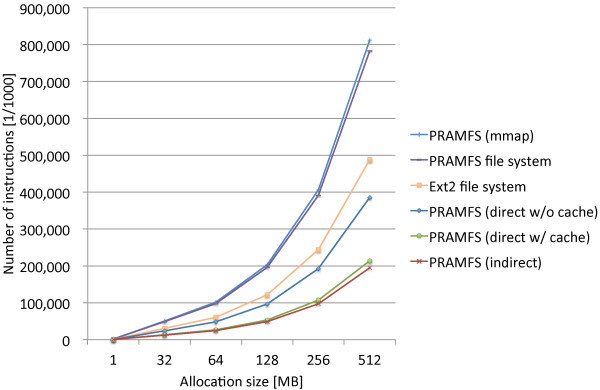
**Comparison of page allocation costs before and after the improvement to the file system by employing the block preallocation cache.**

The block preallocation cache is introduced to the direct method in order to find out its effectiveness. Figure 7 shows the measurement result along with those found in Figure 6 for comparison. PRAMFS (direct w/ cache) is the case that the block preallocation cache is applied to the direct method. We can see that the block preallocation cache can significantly reduce the block allocation cost. The block allocation cost of the direct method with the block preallocation cache is approximately 55% of the cost without the cache, and is only 9.8% more than the cost of the indirect method. While the same amount of the reduction can be expected for the mmap method, the expected result is still not enough to make the mmap method comparable with the direct and indirect methods.

### 5.2 Modification to free block management structure

The first improvement is only effective for the direct method but not enough for the mmap method. In order to further reduce the block allocation costs, we modified the free block management structure of PRAMFS. It originally employs the bitmap data structure to manage its free blocks. As described in Section 4.3, the bitmap data structure works effectively for HDDs while there is no benefit for NV memory because it does not have to take the seek time of HDDs into consideration. Therefore, memory based data structures should work better for NV memory to take advantage of its byte addressability.

In order to verify the above notion, we modified PRAMFS to use a linked list for the free block management. We chose a linked list because of its stability and the least implementation cost. It is well known that a linked list can be used for the free block management of a file system (Bach 1986). Since our objective for this work is to investigate how the management data structures of file systems affect the memory allocation costs, the implementation cost should be saved. Once we find a linked list works effectively, we should consider other more functional data structures for the free block management, such as the buddy system. The Linux kernel provides the standard operations for basic data structures, and the operations for a linked list are included. We used these operations to add and delete a block to the linked list of the free blocks. The region used for the free block bitmap was removed, and is now used for data blocks.

**Figure 8:**
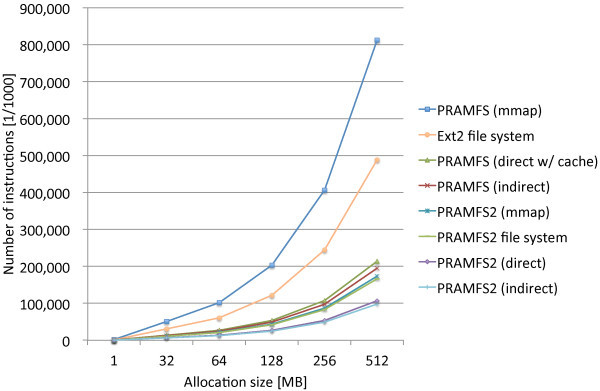
**Comparison of page allocation costs before and after the improvement to the file system by modifying the free block management structure.**

Figure 8 shows the measurement results along with the previous results for comparison. In the figure, PRAMFS2 represents the modified PRAMFS that employs a linked list for the free block management. The modified PRAMFS considerably performs better than the original PRAMFS for all of the measurement results. The indirect method remains the best, and the direct method of the modified PRAMFS comes next with 8.6% overhead. The cost of the mmap method is still 1.8 times as much as that of the indirect method, but is reduced to 21% of the original PRAMFS. Writing to PRAMFS becomes 2.9 times as efficient as Ext2.

The results described above show that the use of a linked list enables the efficient free block management of a file system on NV memory. The bitmap data structure for the free block management is apparently not suitable for a file system on NV memory, and should be avoided. Other more functional data structures are worth consideration. Especially, the buddy system is used for the memory allocator in the Linux kernel, and can handle the memory allocation requests of variable sizes; thus, its applicability to a file system should be investigated.

## 6 Effects on application performance

This section evaluates the effects of the proposed methods on application performance in order to investigate the normal execution behavior of a program.

### 6.1 Evaluation method

Since there are various kinds of applications, there is no single application program that represents all of their workloads. Therefore, a mixed application workload needs to be used for the evaluation. One such example is kernel build of which process involves different kinds of programs, such as a compiler, a linker, and so on, that are executed for numerous times. Therefore, we measured the execution cost of Linux kernel build for each of the proposed method.

The execution environment for the evaluation employs QEMU as the previous sections. The user land programs of CentOS 6.4 are installed on PRAMFS constructed on NV memory. Application programs normally use the malloc library function for memory allocation, and allocated memory spaces are internally managed and reused for future allocation. The direct and indirect methods require no modification to these programs since they are transparent to application programs. The mmap method, however, requires applications to use the mmap system call. In order to avoid the modifications to them, the malloc library function needs to be modified. We modified the malloc library function provided by jemalloc (Evans 2006) because of its excellent performance and clean implementation. In order to make a fair comparison, the unmodified version of jemalloc is used for the measurements of the direct and indirect methods, and the modified version is used for the mmap method. For both of the cases, setting the LD_PRELOAD environment variable enables application programs to use the malloc library function of jemalloc instead of the one provided in the standard library.

### 6.2 Experiment results

**Figure 9:**
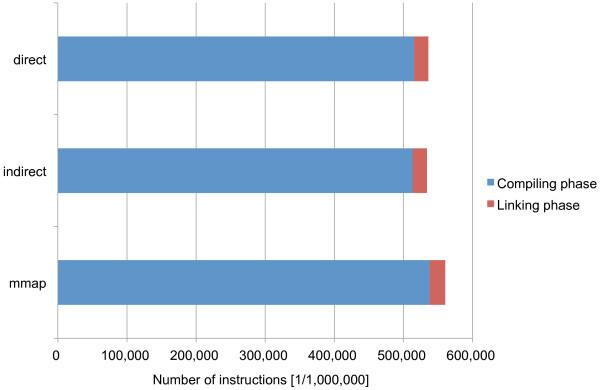
**Performance comparison of the proposed methods by building the Linux kernel.**

Figure 9 shows the results of building the Linux kernel by employing the proposed methods. The cost of building the Linux kernel is divided in the two phases, compiling and linking. The compiling phase mostly consists of compiling C and assembly files to object files. The linking phase basically consists of linking object files to create the bootable Linux kernel. The indirect method performs the best as shown in the previous experiments although its advantage is much smaller. The direct and mmap methods pose 0.4% and 5.0% overheads, respectively, for the total execution times. Because the linking phase requires more memory allocation, the direct and mmap methods pose 0.5% and 10.7% overheads, respectively, that are larger than the total overheads.

The overheads of the direct and mmap methods over the indirect method are much smaller than those measured only for memory allocation. This is because 1) memory allocation is only a part of the processes of compiling and linking files, and 2) the malloc library function usually utilizes allocated memory by recycling freed regions. Therefore, the allocation of main memory pages does not happen so often, so that the performance differences of the proposed methods are mitigated.

## 7 Effects of the access latency of non-volatile main memory

This section evaluates the effects caused by the access latency of NV memory.

### 7.1 Evaluation method

In order to evaluate the effects caused by the longer access latency of NV memory, we performed cycle-accurate full-system simulation by employing MARSSx86 (Patel et al. 2011). MARSSx86 accurately simulates CPU micro architectures and also the latencies of memory hierarchy including cache and main memory. MARSSx86 supports the two micro architectures, Intel Atom and Intel Xeon Westmere. The latencies of DRAM are configured to be 80ns and 55ns for Atom and Xeon, respectively. Table 2 shows the configurations of the target CPUs used for the simulation.

**Table 2:** **Configurations of target CPU micro architectures used for the experiments**

CPU	Intel Atom	Intel Xeon Westmere
Clock	2.13 GHz	3.40 GHz
L1I cache	32 KB	32 KB
L1D cache	24 KB	32 KB
L2 cache	512 KB	256 KB
L3 cache	N/A	8 MB

We modified the memory controller module of MARSSx86 to take into account the different access latency of NV memory. Because MARSSx86 employs QEMU in order to emulate devices, we ported our modifications for QEMU to support NV memory also to MARSSx86, so that the data written in NV memory persist across shutdown and reboot of MARSSx86.

There are different NV memory technologies that have been researched and developed. We use PCM since it is the most popular and closest to mass production. There are several different numbers available as the access latencies of PCM. A number of literatures including (Lee et al. 2009; Qureshi et al. 2009) use 150ns as an estimated write latency. A more recent literature (Jiang et al. 2013) uses 1000ns as the write latency realized by an actual product of PCM from Numonyx. Since there is the significant difference between these latencies, we use both of them and investigate the effects of the different latencies. The all literatures claim that the read latency of PCM is comparable to DRAM; thus, we used the same read latency as DRAM. We also use the same latency as DRAM for comparison.

### 7.2 Experiment results

**Figure 10:**
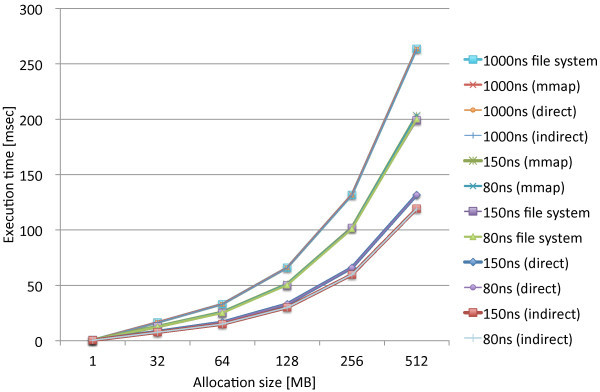
**Page allocation costs measured on the Intel Atom micro architecture simulating the different write latencies of NV memory.**

**Figure 11:**
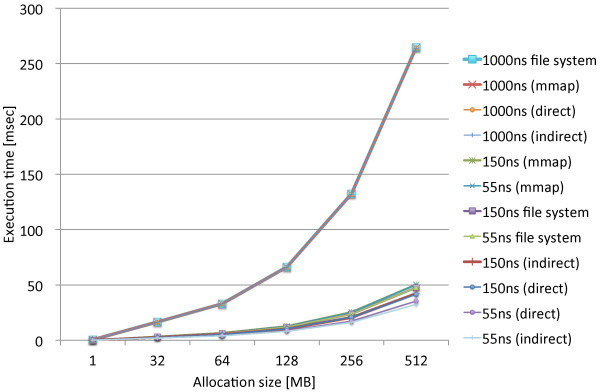
**Page allocation costs measured on the Intel Xeon Westmere micro architecture simulating the different write latencies of NV memory.**

**Figure 12:**
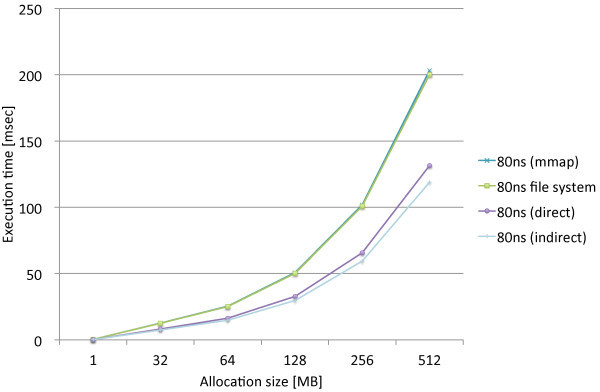
**Page allocation costs measured on the Intel Atom micro architecture simulating the 80ns write latencies of NV memory.**

**Figure 13:**
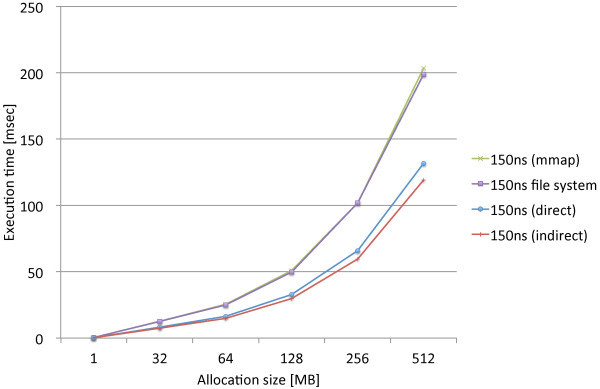
**Page allocation costs measured on the Intel Atom micro architecture simulating the 150ns write latencies of NV memory.**

**Figure 14:**
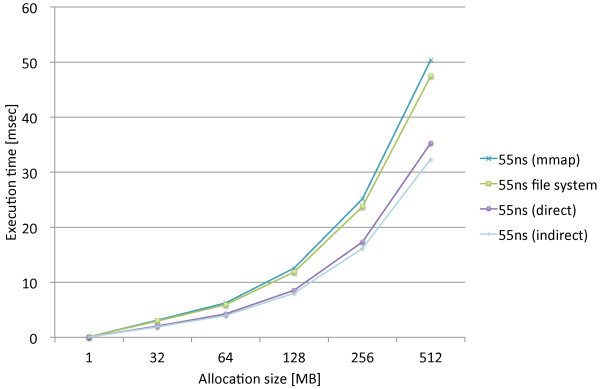
**Page allocation costs measured on the Intel Xeon Westmere micro architecture simulating the 55ns write latencies of NV memory.**

**Figure 15:**
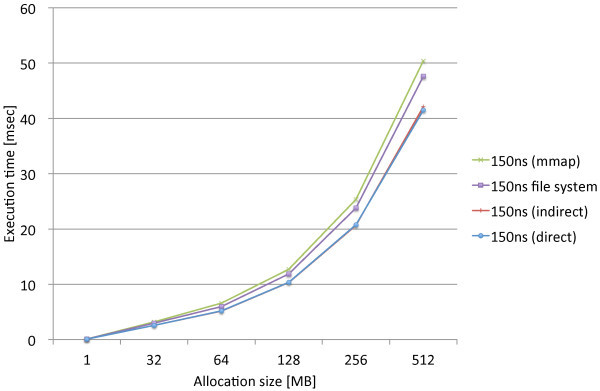
**Page allocation costs measured on the Intel Xeon Westmere micro architecture simulating the 150ns write latencies of NV memory.**

We performed the same experiments that measure the page allocation costs by the three proposed methods, direct, indirect, and mmap, along with the standard file access API. We used the modified file system named PRAMFS2 described in Section 5.2 for the measurements.Figures 10 and 11 show the measurements results including the different write latencies of NV memory for Atom and Xeon, respectively, in order to see the overall differences caused by the different latencies. From the results, we see the results of the 150ns and DRAM latencies do not make remarkable difference on both of the CPUs. While there are slightly larger differences on Xeon, there is approximately no difference on Atom. The integration methods are rather the major causes of the differences. We also see the results of the 1000ns latency are the same for the all methods on both of the CPUs. The 1000ns latency is considered to be long enough to absorb the differences of access methods and CPUs. Moreover, we see that the effects of the different latencies are moderate on Atom while they are much more significant on Xeon. The 1000ns latency does not make the page allocation cost significantly larger on Atom. Its results is only 1.3 times as large as the mmap method and 2.2 times as large as the indirect method. On Xeon, however, the 1000ns latency makes the cost much larger. The results of the 1000ns latency is 5.2 times as large as the mmap method and 8.1 times as large as the indirect method. Although these results are too limited to evaluate the whole performance effects caused by longer write latencies, we estimate that the effects of the 150ns write latency can be amortized within memory hierarchy. The 1000ns write latency, however, causes the apparent effect on performance; thus, it requires aid to cover long latency.Figures 12 and 13 show the measurements results only including the 80ns and 150ns write latencies of NV memory for Atom, respectively, in order to see the differences caused by the different latencies and integration methods more precisely. As described above, the deference between the 80ns and 150ns latencies is very small. The largest difference is 0.15% for the direct method. As the results show almost no difference between the 80ns and 150ns latencies, the differences between the integration methods are also negligible. Interesting information we could obtain from these results is the relevance of the results we can obtain from QEMU. The indirect method performs 9.6% and 7.9% faster than the direct method on Atom and QEMU, respectively. The mmap method performs 54.5% and 62.6% slower than the direct method on Atom and QEMU, respectively. Although the difference is slightly larger for the mmap method, they do not differ completely. Therefore, we should be able to use QEMU in order to estimate the performance on Atom.Figures 14 and 15 show the measurements results only including the 50ns and 150ns write latencies of NV memory for Xeon, respectively. The results between the 50ns and 150ns latencies are much more remarkable on Xeon than Atom. The direct and indirect methods make considerable differences between the 50ns and 150ns latencies while the mmap method and file access API make no difference. The direct and indirect methods perform 17.4% and 30.0% slower, respectively, with the 150ns latency than the 55ns latency. Since the 150ns latency makes the indirect method much slower, it makes the indirect method slower than the direct method. These results are contrary to the inference based on the cache configurations of Atom and Xeon. The sizes of the last level cache (LLC) on Atom and Xeon are 512 KB and 8 MB, respectively. Since Xeon’s LLC is much larger than Atom’s, it is more reasonable that the 150ns latency affects the performance of Xeon less than Atom. It is possible that the out-of-order execution on Xeon is more sensitive to longer latency than the in-order execution on Atom. We need more investigation in detail to understand the effects of longer latency on Xeon.

## 8 Related work

There are only a few papers that describe the integration of main memory and storage. Bailey, et al. (Bailey et al. 2011) discusses various possibilities, including the integration of main memory and storage, made possible by employing NV memory as main memory. Jung, et al. (Jung and Cho 2011) describes the policy and possible effect of the integration. Neither of them, however, realized the integration. This paper describes the methods to realize it and presents their design and implementation in the Linux kernel.

There are a number of researches conducted to enable NV memory to be used as main memory (Lee et al. 2009; Mogul et al. 2009; Qureshi et al. 2009, 2010; Zhang and Li 2009; Zhou et al. 2009). Since these are the researches of the computer architecture to integrate NV memory into main memory by overcoming its limitations, there is no consideration to integrate main memory and storage. On the other side, there are the researches that construct file systems on NV memory by taking advantage of its byte addressability (Condit et al. 2009; Wu and Reddy 2011). Although these researches utilize the feature that enables NV memory to be used as main memory, they do not consider NV memory as main memory at all.

FlashVM (Saxena and Swift 2010) and SSDAlloc (Badam and Pai 2011) propose the methods to extend usable memory spaces virtually larger by utilizing SSDs and making page swapping faster than the existing mechanism. While these improve the virtual memory system of the OS kernel, main memory and storage remain separated.

NV-Heaps (Coburn et al. 2011) introduces a persistent object system that specifically targets NV memory. Application programs use them to persistently maintain objects safely and consistently on NV memory. Whole-system persistence (WSP) (Narayanan and Hodson 2012) proposes a system, of which memory is NV memory only. The paper categorizes systems with NV memory as block-based, persistent heaps, and WSP. WSP provides applications with a view that all objects are persistent, and it employs a transparent mechanism to continue its operation on power failure. While these papers propose novel approaches to use NV memory from applications, they do not discuss a means for the kernel to manage NV memory where persistent objects are stored. Our approach, which uses a file system as a base to manage NV memory, addresses the memory management within the OS kernel. It is orthogonal to them; thus, it can be used to store persistent objects on a file system and to enable their protection and sharing.

Byte-addressable persistent RAM APIs (Febiansyah and Kwon 2013) discusses the APIs that address wear leveling, which is required by some types of NV memory. While wear leveling is an important issue to support NV memory, it is outside the scope of this paper since our focus of this paper is to propose the methods that can integrate the management of main memory and storage. It is possible for a file system to support a certain level of wear leveling as we can find it in YAFFS (ALEPH ONE 2006), it is one of our future work.

## 9 Summary and future work

This paper presented the integration methods of the main memory and file system management for NV memory, so that it can be used as both main memory and storage. The presented methods use a file system as their basis for the NV memory management; thus, the internal data structures of a file system have impacts upon the performance of the integration methods. We implemented the proposed methods in the Linux kernel, and performed the evaluation on a system emulator. We performed the evaluation in three phases. We analyzed the first preliminary experiment results and devised the improvements. The second experiment results showed that 1) the proposed methods can perform comparably to the existing DRAM memory allocator and significantly better than the page swapping, 2) their performance is affected by the internal data structures of a file system, and 3) the data structures appropriate for traditional HDDs do not always work effectively for NV memory. These results were shown by concrete experiments with the implementation in the Linux kernel. Finally, we performed the evaluation of the effects caused by the longer access latency of NV memory by cycle-accurate full-system simulation. The results showed that the effect on page allocation cost is limited if the increase of latency is moderate.

Although we performed simulation by employing a cycle-accurate full-system simulator that were modified to take into account the different access latency of NV memory, the results are rather contrary to the inference based on the configurations of simulated CPUs. We need more investigation in detail to understand the effects of longer latency.

## Endnote

^a^ XIP is the abbreviation of eXecution In Place.
